# Predictive factors associated with adjacent teeth root resorption of palatally impacted canines in Arabian population: a cone-beam computed tomography analysis

**DOI:** 10.1186/s12903-022-02249-4

**Published:** 2022-06-03

**Authors:** Hana’a A. Al-Kyssi, Naela M. Al-Mogahed, Zainab M. Altawili, Faiz N. Dahan, Abeer A. Almashraqi, Khalid Aldhorae, Maged S. Alhammadi

**Affiliations:** 1 Department of Orthodontics, Pedodontics and Preventive Dentistry, Faculty of Dentistry, Sana’a University, Sana’a, Republic of Yemen; 2 Faculty of Dentistry, Jordan University of Science and Technology, Irbid, Jordan; 3 College of Dentistry, Thamar University, Thamar, Republic of Yemen; 4 Department of Pre-Clinical Oral Health Sciences, College of Dental Medicine, QU Health, Qatar University, Doha, Qatar; 5 Department of Orthodontics, College of Dentistry, Thamar University, Thamar, Republic of Yemen; 6 Department of Orthodontics, College of Dentistry, University of Ibn Al-Nafis for Medical Sciences, Sana’a, Republic of Yemen; 7 Orthodontics and Dentofacial Orthopedics, Department of Preventive Dental Sciences, College of Dentistry, Jazan University, Jazan, Saudi Arabia

**Keywords:** CBCT, Lateral incisors, Palatal canine impaction, Root resorption

## Abstract

**Background:**

This study aimed to evaluate three-dimensionally the factors associated with adjacent teeth root resorption of palatally impacted canines.

**Methods:**

In this retrospective cross-sectional study, one-hundred and fourteen cone beam computed tomography scans with palatally impacted maxillary canines were evaluated for the presence of adjacent root resorption. Seven parameters were analyzed: alignment of maxillary incisors, presence of deciduous canines, first premolars’ roots configuration, impacted canines rotation, angulation of impacted canine to the midline, contact relationship, and area of contact with adjacent teeth. The association between dependent and independent qualitative and quantitative variables was analyzed using chi-square and independent student’s t-test, respectively. The multivariate analysis was performed using regression analysis. The significant value was set at *P* ≤ 0.05.

**Results:**

The overall incidence of vertical, horizontal impaction and adjacent root resorption were 92, 8 and 77.2%, respectively. The apical third was the most involved area (57%); resorption of a single tooth was found in 21.9% of the total sample. The most common resorbed teeth were lateral first premolars (24.6%), followed by central lateral incisors (20.2%), and lateral incisors (15.8%) of the total sample. The severity of resorption was highest in grade I (31.5%) and lowest in grade III (7.6%). Three variables showed significant differences between resorption and non-resorption groups namely; canine rotation (*P* < 0.013), contact relationship (*P* < 0.001), and area of contact with adjacent teeth (*P* < 0.001). Regression analysis revealed an association between adjacent root resorption and permanent canine rotation, adjacent premolars’ roots configuration, contact relationship, and area of contact (*P* < 0.05).

**Conclusion:**

Two-thirds of impacted maxillary canines showed a form of root resorption. The most commonly resorbed tooth was the lateral incisors while the least affected one was the central incisors with apical one-third being of the highest risk. The predisposing factors including the canine rotation, premolar with separated roots, contact relationship, and area of contact with adjacent teeth are to be considered for any interceptive treatment.

## Introduction

Eruption of teeth is a complex process, therefore early, delayed or even failure of teeth eruption may occur. Once the scheduled time of teeth eruption passed, these teeth considered as an impacted teeth. Third molars are the most commonly impacted teeth, followed by permanent canines [1]. The exact etiology of teeth impaction is unknown; several etiological factors for canine impactions have been proposed: localized, systemic, or genetic factors; the most common localized factor is arch length-tooth size discrepancy [2]. Two main theories associated with displaced maxillary canines are the genetic theory and guidance theory [1]. Guidance theory assumed that maxillary canines need a guide to erupt. Any guiding path disturbance will complicate the eruption process. Whereas genetic theory suggests that there is nothing to do to control canine eruption as it is a pre-programmed process [3–5].

Nevertheless, these theories give an idea about the local factors that may be controlled during early age. The displacement may cause occlusal disturbances and/or abnormalities to adjacent structures. The majority of maxillary canine impactions were close to the adjacent teeth and might be a source of root resorption [6–9]. The overall incidence of palatally impacted maxillary canines is 56.99 and 56.87% are associated with adjacent teeth root resorption [7, 10]. The presence and extent of root resorption of the adjacent teeth may complicate the orthodontic treatment plane. Long term follow up studies reported that removal or orthodontic traction of the impacted maxillary canine might stop resorption progression; it is likely that restitution of protecting tissues around the small to moderate defect have a good prognosis, while teeth with severe resorption involving the pulp are less amenable to repair [11, 12].

It was assumed that there are several risk factors may be predictive to increase the risk of resorption when the maxillary canine is impacted and/or displaced. Several studies have focused on these factors by evaluating radiographic changes in the intergrity of the surrounding roots related to the site of impaction [7, 13–17]. The main evaluated factors were impacted maxillary canine rotation [14], the configuration of the first premolars’ roots, contact location, contact relationship [7, 16], over-retained deciduous canine, loss of arch space and inclination of an impacted tooth [13, 15]. However, these can be managed early for a good orthodontic treatment prognosis. The interceptive treatment seemes to be the most convenient approach [18–20].

Adjacent root resorption is a possible side effect in most cases, the most commonly affected tooth is mostly the maxillary lateral incisors [6, 10, 21]. The detection of such complications was established by using different methods of the dental radiograph. Haney et al. [22] evaluated the accuracy of cone beam computed tomography (CBCT) compared to orthopantomogram; CBCT can give details in three planes of space; coronal, sagittal, and axial planes. Numerous studies used CBCT to detect resorption of teeth adjacent to palatal impacted canine [6, 16]; Kalavritinos et al. [16] examined 61 cases and found canine impaction caused lateral root resorption only in 18.5%, while Alassiry et al. [6] evaluated 169 subjects for impacted maxillary canines and reported 74.3% of lateral root resorption. The location of resorption at the apex, middle, or cervical has also been reported; Alemam et al. [7] found that palatally impacted canine affected 74% of lateral incisors, and the most affected part was at the midle and apical thirds in 89%.

This study aimed to three-dimensionally investigate the factors associated with adjacent teeth root resorption of palatally impacted canines as a primary outcome and to assess the distribution of maxillary impacted canines in a sample of Arabian population as a secondary outcome.

## Materials and methods

### Study design

This is a retrospective cross-sectional study. The study sample included patients seeking dental treatment aged between 18 and 30 years who were admitted to the oral and maxillofacial radiology center, Sana’a, Republic of Yemen, from August 2017 to January 2019.

### Study sample size

The sample size was selecetd to be greater than the previous studies having the same aim and study design [16, 23–25]. The inclusion criteria were: (1) age range between 18 and 30 years old, (2) presence of maxillary impacted canines, and (3) full set of permanent teeth except for the third molars. The exclusion criteria inluded; (1) history of trauma to the teeth and/or bone fractures, (2) history of metabolic disorders, (3) craniofacial syndromes or anomalies that could affect head and neck regions, (4) presence of a pathological lesion/s in the craniofacial area (5) history of previous orthodontic treatment, and (6) bad quality CBCT images.

### Three-dimensional imaging

The CBCT images were evaluated in the orthodontics and dentofacial orthopedics department at the College of Dentistry, Thamar University, Republic of Yemen. The CBCT data were obtained from the CBCT machine (Pax-flex3DP2, Vatech, Korea). The CBCT images were taken with the following parameters; the field of view, 15 × 15 cm; 95 kV; 5.8–7.1 mAs; and 15 s exposure time. The selected voxel dimension was 0.3 mm, and the slice thickness was 2 mm. Frankfort horizontal plane was parallel to the floor guided by crossing laser guide. According to the imaging protocol, the patient was instructed not to swallow or move during the scanning process.

**Fig. 1:**
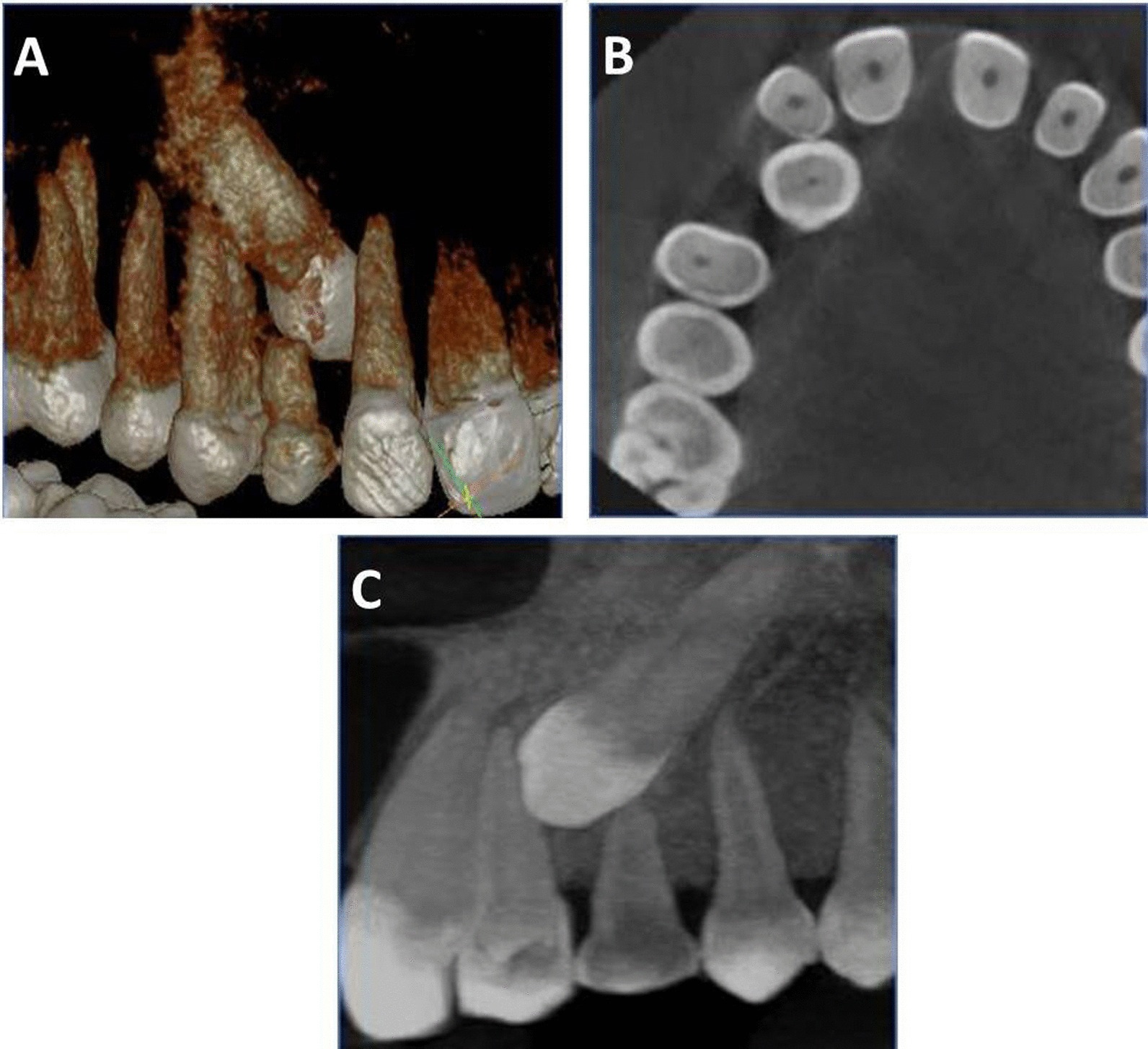
Three dimensional views generated from Ez3D plus software: **A** and **B** cross-sectional view showing contact relation of impacted 13–12, **C** position of the impacted canine in relation to the maxillary lateral incisor and first premolar

All analysis was performed using an integrated specialized software program (Ez3D plus^®^ Vatech, Korea) (Fig. 1).

### Outcome assessment

**Fig. 2:**
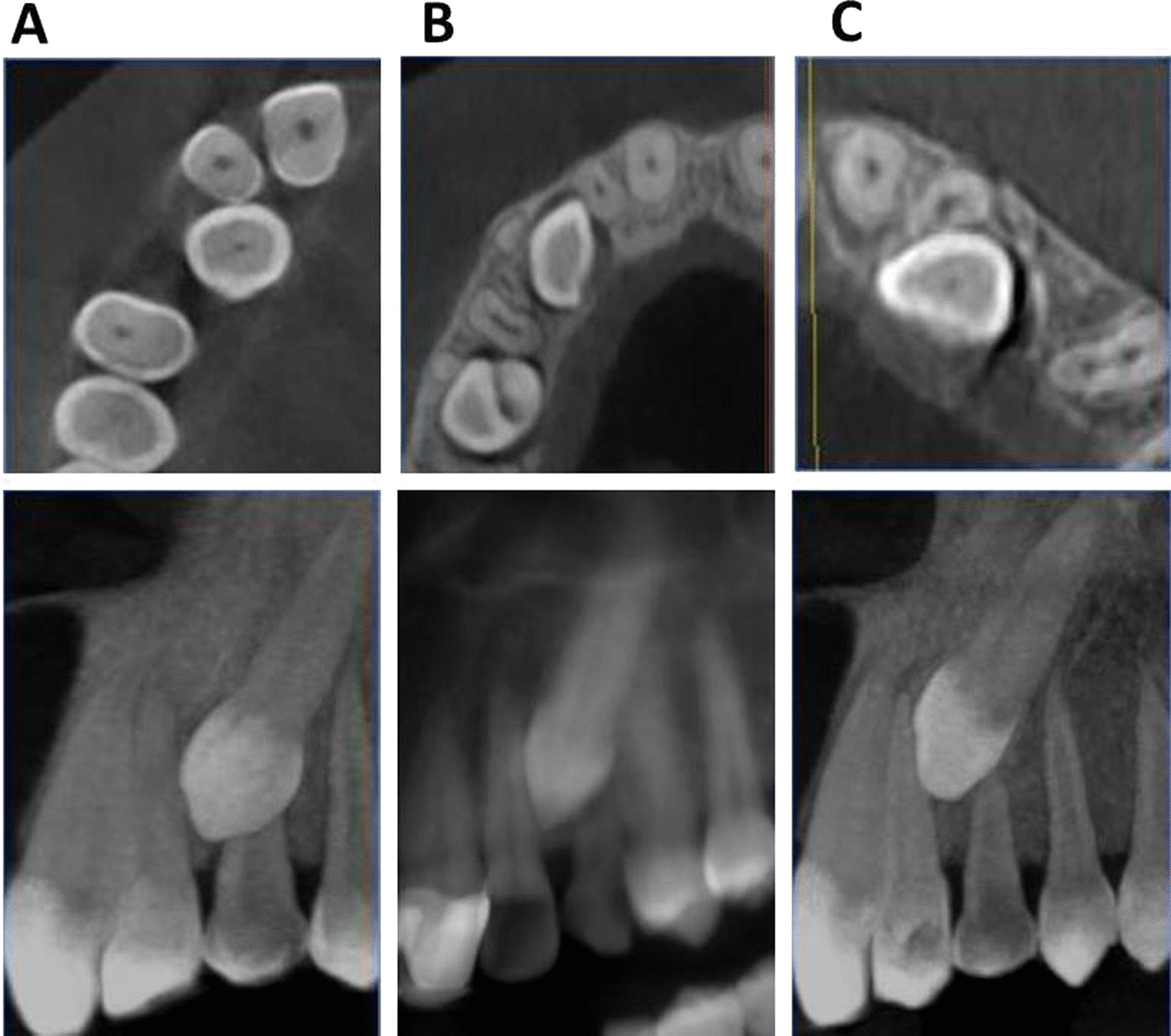
Severity of lateral root resorption according to Ericson and Kurol grading system; **A** grade 0, **B** grade 1, and **C** grade 2 root resorption

The root resorption grading was evaluated using Ericson and Kurol’s classification, (Fig. 2) [26]:

- Grade 0: Intact root surfaces except for the loss of cementum.
- Grade 1: Slight resorption, up to half of the dentine thickness to the pulp.
- Grade 2: Moderate resorption, halfway to the pulp or more; the pulp is covered with dentine.
- Grade 3: Severe resorption; the pulp is exposed.

**Fig. 3:**
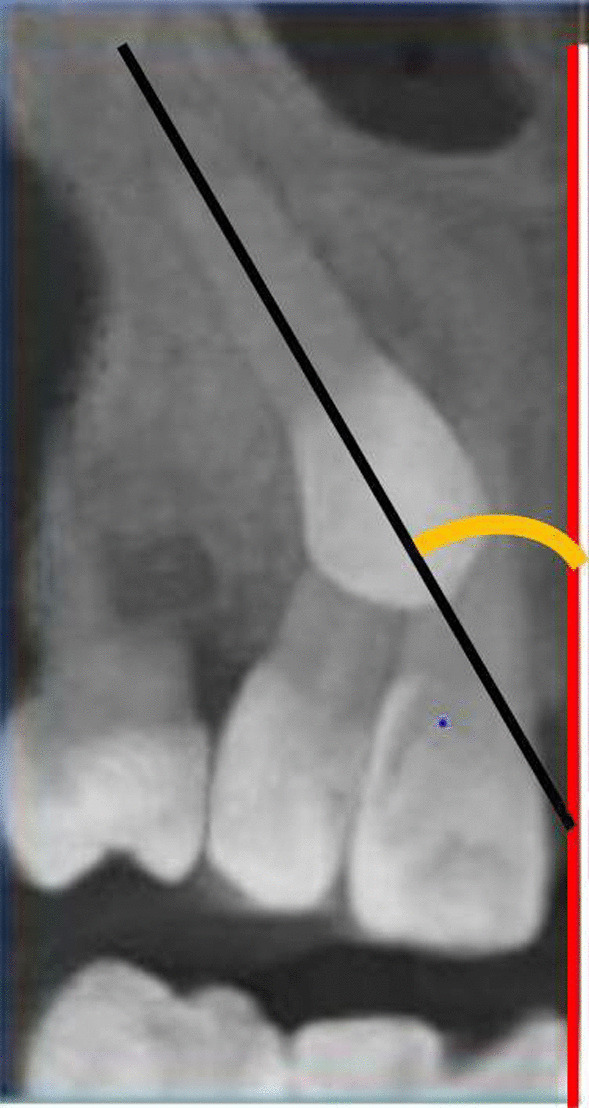
CBCT image illustrating the angular measurement reference of maxillary impacted canine to the midline

The prediective factors for root resorption of palatally canine impaction was assessed according to the following criteria:

- Alignment of the permanent upper incisors (well-aligned, spaced, or crowded).
- Presence/missing of maxillary deciduous canine with/without root resorption.
- According to Walker et al. [24], the direct contact of the impacted maxillary canine with adjacent teeth was measured as the shortest distance between the impacted canine and the adjacent incisor. Contact was defined as proximity of less than 0.5 mm.
- The contact location was classified as the cervical, middle, or the apical third of the root of the adjacent tooth.
- For canine angulation to the midline, the angle between the long axis of the canine and the midline between central incisors was measured. It was categorized according to the severity into mild (0–15°), moderate (15–30°), and severe (> 30°) (Fig. 3) [15, 24].

A random sample of 20 CBCT cases were re-evaluated in 1-month intervals by the same evaluator (H.A.) and by another evaluator (A.A.) to test the intra- and inter-examiner reliability analysis of the assessed measurments.

### Statistical analysis

The collected data was analyzed using the Statistical Package for the Social Sciences (SPSS) program (Version 24; Inc., Chicago. IL, USA). The reliabilty analysis was performed using Kappa statistics and the intra-class correlation cofficient (ICC). The association between dependent and independent qualitative and quantitative variables was analyzed using chi-square and independent student’s t-test, respectively. The multivariate analysis was performed using regression analysis. A *P* < 0.05 was considered to be significant.

## Results

The study sample included 114 subjects. The reliability analysis was excellent and it was as low as 0.836 for canine angulation to the midline to as high as 1.00 for other variables. The mean age of the included subjects was 23.32 ± 4.18 years.

The sample consists of 40 males (35.1%) and 74 females (64.9%). The side of impaction showed that 56 (48.7%) impacted canines located on the right and 58 (51.3%) on the left side (Table 1). Overall, 106 palatally impacted canines were in the vertical position (92%), while 34 were in the horizontal position (8%).The primary canine was present in 54.8% of selected subjects. Amongst them, 40.9% show signs of physiological resorption, whereas 13.9% did not undergo physiological resorption. The separated root of the first premolar was predominant in 53% (Table 1).

**Table 1:** Distribution of maxillary impacted canines and root resorption in different categories

Variable	Frequency	Percent
*Gender*		
Male	40	35.1
Female	74	64.9
*Side of impaction*		
Right	56	48.7
Left	58	51.3
*Incisor’s alignment*		
Spacing	14	12.2
Aligned without space	96	83.5
Crowding	4	3.5
*Maxillary deciduous canine*		
Missed	51	44.8
Present with resorption	47	41.2
Present without resorption	16	14
*Canine rotation*		
Mesiopalatal	57	50
Distopalatal	57	50
*Configuration of first premolar root*		
Single	11	9.6
Separated	61	53
Fused	42	36.5
*Type of impaction*		
Vertical	106	92.2
Horizontal	8	7
*Resorption*		
No resorption	26	22.8
Resorption	88	77.2
*Affected teeth*		
Lateral incisors	18	20.5
First premolars	6	6.9
Central incisors	1	1.1
Central and lateral incisors	23	26.1
Lateral incisors and first premolars	28	31.8
Central incisors, lateral incisors, and first premolars	12	13.6
*Severity of resorption*		
Grade 0	33	25.3
Grade 1	41	31.5
Grade 2	30	23
Grade 3	10	7.6

As presented in Table 1, the overall prevalence of root resorption was 77.2%. Root resorption presented mostly in the lateral incisors (20.5%), the first premolars (6.9%), the central incisors (1.1%), the central and lateral incisors (26.1%), the lateral incisors and first premolars (31.8%), and the central incisors, lateral incisors, and first premolars simultaneously (13.6%). The severity of resorption was highest in grade I (31.5%) and lowest in grade III (7.6%). Distributions of contact relation, location of contact, resorbed teeth and the severity of resorption between the right and left sides are presented in Table 2.

**Table 2:** Distributions of contact relation, location of contact, resorbed teeth and the severity of root resorption between the right and left sides

Variable	Right	Left	Right	Left
	Frequency		Percent	
*Contact relation*				
Central incisors	1	0	100	0
Lateral incisors	7	11	38.89	61.11
First premolars	5	1	83.33	16.67
Central and lateral incisors	12	11	52.17	47.83
Lateral incisors and first premolars	16	2	88.89	11.11
Central incisors, lateral incisors, and first premolars	6	6	50	50
*Location of contact*				
Cervical lateral incisors	1	2	33.33	66.67
Middle lateral incisors	10	10	50	50
Apical lateral	19	9	67.86	32.14
Middle central, apical lateral incisors	1	4	20	80
Middle lateral incisors, apical premolars	13	11	54.17	45.83
Middle central, lateral incisors and apical first premolar	4	4	50	50
*Resorption*				
Central incisors	1	0	100	0
Lateral incisors	7	11	38.89	61.11
First premolars	5	1	83.33	16.67
Central and lateral incisors	12	11	52.17	47.83
Lateral incisors and first premolars	16	12	57.14	42.86
Central incisors, lateral incisors, and first premolars	6	6	50	50
*Severity of resorption*				
Grade 0	14	19	42.42	57.58
Grade 1	20	21	48.78	51.22
Grade 2	17	13	56.67	43.33
Grade 3	5	5	50	50

The results of chi-square test with and without root resorption of different studied predictors showed that three measurements were statistically significant, the canine rotation, contact relationship and area of contact. Mesiopalatal and distopalatal rotation was found in 87.7 and 66.7% in the resorption group compared to 12.3 and 33.3% in the non-resorption group, respectively. There was significant difference between the resorption and non-resorption groups in term of the contact relation for all contacted teeth except the central incisors; the middle and apical thirds being significantly the highest portions to be affected by root resorption (Table 3).

**Table 3:** Results of chi-square test with and without root resorption of different studied predictors

Variable	Category	With resorption No. (%)	Without resorption No. (%)	*P* value
Alignment of incisors	Spaced	8 (57.1)	6 (42.9)	0.099
	Aligned	76 (79.2)	20 (20.8)	
	Crowded	4 (100)	0 (0)	
Maxillary deciduous canine	Extracted	43 (84.3)	8 (15.7)	0.157
	Present with resorption	35 (74.5)	12 (25.5)	
	Present without resorption	10 (62.5)	6 (37.5)	
Root morphology of first premolar	Single	11 (100)	0 (0)	0.078
	Separated	48 (78.7)	13 (21.3)	
	Fused	29 (69)	13 (31)	
Canine rotation	Mesiopalatal	50 (87.7)	7 (12.3)	0.013
	Distopalatal	38 (66.7)	19 (33.3)	
Contact relationship	No contact	0 (0)	25 (100)	0.001
	With central incisors	1 (50)	1 (50)	
	With lateral incisors	18 (100)	0 (0)	
	With first premolars	6 (100)	0 (0)	
	With central and lateral incisors	23 (100)	0 (0)	
	With lateral incisors and first premolars	28 (100)	0 (0)	
	With centrals, laterals incisors and first premolars	12 (100)	0 (0)	
Area of contact	No contact	1 (3.8)	25 (96.2)	0.001
	Cervical lateral incisors	2 (66.7)	1 (33.3)	
	Middle lateral incisors	20 (100)	0 (0)	
	Apical lateral incisors	28 (100)	0 (0)	
	Middle central, apical lateral incisors	5 (100)	0 (0)	
	Middle lateral incisors, apical first premolar	24 (100)	0 (0)	
	Middle central, lateral incisors and apical first premolar	8 (100)	0 (0)	

The angular measurements between the long axis of the canine and the midline showed that 10.2, 27.3, and 62.5% of the resorption cases were mild, moderate and severe, respectively. There was no statistical difference between these measurements between the resorption and non-resorption cases (Table 4).

**Table 4:** Results of student t-test on the number and percentage of the canine angulation categories to the midline with the incidence of root resorption

	Mild (0–15°)	Moderate (16–30°)	Severe (> 30°)	Total%
Root resorption	9 (10.2%)	24 (27.3%)	55 (62.5%)	88 (100%)
No resorption	2 (7.7%)	7 (26.9%)	17 (65.4%)	26 (100%)
*P* value	> 0.05			

The Regression analysis revealed an association between adjacent root resorption and permanent canine rotation (*P* = 0.037), adjacent premolar root configuration (*P* = 0.020), contact relationship (*P* = 0.009), and area of contact (*P* = 0.001) (Table 5).

**Table 5:** Results of regression analysis between the root resorption and studied predictors

Variable	OR	*P* value	95% Confidence interval	
			Lower	Upper
Canine rotation	2.880	0.037	1.066	7.785
Alignment of incisors	0.390	0.170	0.102	1.499
Maxillary deciduous canine	0.344	0.088	0.101	1.171
Root morphology of first premolar	5.327	0.020	1.307	21.722
Contact relationship	0.084	0.009	0.013	0.542
Area of contact	1.458	0.001	1.216	1.748

## Discussion

The morbidity that may arise from palatally impacted canine ranged from the adjacent root resorption to a more destructive effect like compound-odontoma like lesion [27]. Hence, many studies designed to formulate and investigate methods to inhibit this complication by controlling several predisposing factors; these studies found a high association between these factors and the adjacent root resorption [6, 7, 10].

In line with that, several studies evaluated ethnic groups as a predisposing factor and concluded that different ethnic groups have different incidences of root resorption in palatally impacted canines [6, 16]. CBCT is considered the gold standard for localization of impacted canine and its side effects including root resorption and recommended as the most useful method to detect root resorption by the American Academy of Oral and Maxillofacial Radiology [23, 28]. So this study gives a deep insight into the impact of the Arabian ethnic group on the incidence of impaction and root resorption of adjacent teeth for palatally displaced impacted canines by employing CBCT technology.

Alignment of incisors was an important factor for increasing the incidence of canine impaction, only 12.2% of our sample having spaced anterior teeth. This agrees with Montes-Díaz et al. [29], who found that canine palatal impaction was significantly associated with less arch space. In the current study, 55.2% of the sample showed persistence of primary canine, indicating that more than half of our subjects benefited from early intervention. A recent systematic review revealed that if the primary canine barely or does not undergo physiological resorption, an interceptive extraction might benefit and decrease the risk of canine impaction [13]. The configuration of the first premolar showed a high link to canine impaction in the present study; 53% of impaction was associated with a separated root configuration. This is in line with Cao et al. [30], who found that separated roots have a higher incidence of impaction.

There is a wide range of root resorption associated with impacted canines, in this study, 77.2% adjacent root resorption was detected. However, this percentage is close to that reported by Alemam et al. [7], who report an incidence of 74% in the Jordanian population; it was reported as 50.7% in Chinese [21], 77.8% in American [31], and 48.2% in French population [25]. These differences are due to diversity among the ethnic group selected in all mentioned samples. The severity of resorption is mostly reported in mild grades; grade 1 and 2 [6, 17, 21]. The severity of resorption was highest in grade I, this is less than that reported in Saudi (55%) [6], Chinese (46.6%) [21], and French (71.7%) population [17].

In the present study, root resorption was highly associated with contact between impacted canine and other adjacent teeth. This may be because of the eruption force exerted by the canine into the adjacent tooth [32]. Root resorption of the lateral incisors only was 20.5% in the current study; resorption of this tooth was reported to be as low as 19.9% by Kalavritinos et al. [16] to as high as 74% reported by Alemam et al. [7]. For the central incisors only, it was found to be 1.1%, this is the least reported percentage in the literature; it was reported to be as low as 2.3% by Oberoi et al. [33] and as high as 24.7% as reported by da Silva Santos et al. [34] These contradictions reveal the necessity to expand the scope of study to more suspected factors other than canine impaction.

Canine rotation considered as a risk factor for root resorption, this was showed on comparing the resorption and non-resorption groups and also an association was found between this factor and the root resorption. This is in agreement with Alqerban et al. [14], this is because that the mesio-palatal direction was the most commonly encountered. In line with Dekel et al. [35] who concluded that the crowns were mesiobuccally rotated compared with the controls. The difference was higher when the palatally impacted canines were closer to the mid-palatal plane, more anteriorly displaced, thus closer contact with the lateral incisor roots, and high in the alveolus.

Regarding canine angulation to the midline, it was not statistically associated with root resorption. This is in line with Liu et al. [21] and Lai et al. [36], who reported insignificant association with the amount of lateral incisor resorption on CBCT. These indicated that the contact with adjacent teeth is more important to the canine angulation itself.

While more than eight countries share the Arabian ethnic group; nevertheless, the distribution of this ethnicity into different geographical parts exposed to variable environments has a significant impact. Here, we highlight two other similar studies in Saudi Arabia and Jordan. The former has a desert environment and a more developed lifestyle, with large cities, while the latter has a cold mountain, agriculture environment mixed with more developed centers. Yemen's environment is quite diverse between desert, mountain, and valley environments, with poor civil life [37].

Although it was reported in a recent systematic review that the CBCT imaging is less serious and more accurate than the 2D images in evaluating the root resorption [38], the advent of high-performance software makes the use of the superimposition option a valid tool for investigating the effect orthodontic treatment on the root surfaces [39]. Another emerging method is the use of cinematic rendering in CBCT which allows for detailed visualization of surface structures, their plasticity, and 3D configuration [40]. Further studies are recommended to have high-level evidence on the use of these technologies in a detailed assessment of root resorption.

The limitations of this study included; the study didn’t include other ethnic groups in the Arabian region, the sample was limited mainly to the palatally impacted canines, larger sample with labially impacted one is to be considered. The retrospective nature of the study preventing assessment of teeth vitality especially those with considerable amount of resorption.

## Conclusions

- Leaving impacted canines without proper intervention resulted in serious root damage of the adjacent teeth in more than two thirds of the suspected cases with lateral incisors being the most commonly affected tooth.
- The permanent canine rotation, separated root configuration of the first premolar, contact relationship and area of contact with adjacent teeth are predisposing factors for adjacent root resorption.
- Early detection of palatally impacted canine and evaluation of the risk factors for root resorption together with proper interceptive treatment can serve as an alternative of a complicated future treatment.

## Data Availability

The dataset used and/or analyzed during the current study is available from the corresponding author on reasonable request.
